# Cultural and Contextual Adaptation of Digital Health Interventions: Narrative Review

**DOI:** 10.2196/55130

**Published:** 2024-07-09

**Authors:** Aila Naderbagi, Victoria Loblay, Iqthyer Uddin Md Zahed, Mahalakshmi Ekambareshwar, Adam Poulsen, Yun J C Song, Laura Ospina-Pinillos, Michael Krausz, Mostafa Mamdouh Kamel, Ian B Hickie, Haley M LaMonica

**Affiliations:** 1 Brain and Mind Centre The University of Sydney Sydney NSW Australia; 2 Department of Psychiatry and Mental Health Faculty of Medicine Pontificia Universidad Javeriana Bogota Colombia; 3 Department of Psychiatry University of British Columbia Vancouver, BC Canada; 4 Department of Psychiatry Tanta University Tanta Egypt

**Keywords:** cultural adaptation, digital health, context, translation, participatory research, mobile phone

## Abstract

**Background:**

Emerging evidence suggests that positive impacts can be generated when digital health interventions are designed to be responsive to the cultural and socioeconomic context of their intended audiences.

**Objective:**

This narrative review aims to synthesize the literature about the cultural adaptation of digital health interventions. It examines how concepts of culture and context feature in design and development processes, including the methods, models, and content of these interventions, with the aim of helping researchers to make informed decisions about how to approach cultural adaptation in digital health.

**Methods:**

Literature searches for this narrative review were conducted across 4 databases. Following full-text article screening by 2 authors, 16 studies of interventions predominantly focused on the self-management of health were selected based on their detailed focus on the process of cultural adaptation. Key considerations for cultural adaptation were identified and synthesized through a qualitative narrative approach, enabling an integrative and in-depth understanding of cultural adaptation.

**Results:**

The literature demonstrates varying approaches and levels of cultural adaptation across stages of intervention development, involving considerations such as the research ethos orienting researchers, the methodologies and models used, and the resultant content adaptations. In relation to the latter, culturally appropriate and accessible user interface design and translation can be seen as particularly important in shaping the level of adaptation.

**Conclusions:**

Optimizing cultural adaptation involves linking culture with other contextual factors such as economic conditions and social systems to ensure accessibility and the sustained use of digital health interventions. Culturally humble approaches that use the involvement of a broad range of participants, experts, and other stakeholders are demonstrated to spark vital insights for content development, implementation, and evaluation.

## Introduction

### Background

Digital health interventions have increased exponentially in recent years as a means of expanding access to health information and services, improving cost-effectiveness, and empowering people to take control over their health [1]. Decades-long debates around cultural context in public health interventions [2] are now occurring within the digital health literature with the development of culturally appropriate web- and mobile-based interventions. The cultural adaptation of existing interventions is reported to be a cost-effective and efficient way of delivering evidence-based interventions at scale [3], with emerging evidence indicating that health outcomes are more favorable when interventions are responsive to the cultural and socioeconomic context of their intended audience [4-6]. Despite the demonstrated effectiveness, there remains a general lack of guidance about how to approach the cultural adaptation of digital health interventions, which can result in poor adaptations that are unable to effectively engage their intended audiences [7].

Cultural adaptation is often viewed as a systematic process whereby intervention components identified as *cultural* are isolated and tailored to target populations, while adherence to evidence-based components is uncompromised [8,9]. In complex system thinking, however, context is not a mere backdrop to intervention implementation but actively influences intervention success [10]. Adaptation must therefore be part of digital health intervention design from the outset and not a secondary process [11]. Resnicow et al [2] have distinguished between surface and deep structure adaptation to conceptualize the depths of cultural sensitivity in public health interventions, which are also applicable in the development of digital interventions. Cultural sensitivity at a surface structure level involves the integration of observable social and behavioral characteristics among a target population, such as common places, languages, clothing, and health behavior as well as appropriate channels or settings for the delivery of messages and programs [2]. The deep structure dimension of intervention sensitivity is about the factors influencing health behaviors among different racial and ethnic populations. Deep structure adaptation is recommended when an in-depth understanding is required around matters such as how members of the target population perceive the cause, course, and treatment of illnesses. This often involves qualitative research into the ways religion, family, society, economics, and the government influence health [2].

### Objective

There are no 2 cases of cultural adaptation that are alike, and documentation and reporting differ widely. This narrative review collates diverse studies on the cultural adaptation of web- and mobile-based interventions, with a focus on case examples that provide a thorough record of the stages and processes involved. The aims of this review are to (1) analyze different approaches and levels of cultural adaptation in the digital health literature and (2) examine how the concepts of culture and context feature in the methods, models, and content of these interventions. To date, systematic reviews have emphasized the effectiveness of culturally adapted digital health interventions relative to those interventions that are left unadapted [5,6]. Therefore, through a qualitative narrative synthesis of learnings about major aspects of cultural adaptation, this review offers a distinctive and novel perspective that can be used to inform decision-making in the development of culturally appropriate web- and mobile-based interventions.

## Methods

### Search Strategy

The narrative review approach used here aims to provide a scholarly summary and interpretation of the digital health literature, with the purpose of synthesizing and deepening the understanding of cultural adaptation in digital health. The intention is not to present a hybrid systematic narrative review or evaluate the validity of a particular method or intervention [12]. We have prioritized relevant texts to highlight distinctive contributions and major themes, rather than presenting an exhaustive overview of the entire body of literature with strict inclusion and exclusion criteria [13]. By focusing on qualitative interpretation and synthesis of the literature, our narrative review differs from, but complements, a quantitative approach [14].

The eligibility criteria for this review included (1) studies of the design, development, or evaluation of a digital health intervention for the purposes of self-management of health and (2) studies that included a detailed description of how the digital intervention had been adapted to be culturally and contextually appropriate for target end users. We conducted literature searches across 4 databases, including CINAHL, PsycINFO, Scopus, and Ovid MEDLINE, as well as 2 separate hand searches between October and November 2022. The search was limited to peer-reviewed, English language papers. The search terms included (“digital health” OR “digital intervention” OR “mHealth” OR “mobile health” OR “eHealth” OR “telemedicine” OR “telehealth” OR “parenting app” OR “parent* app”) AND (“sociocultural factors” OR “cultural sensitivity” OR “cultural adaptation” OR “foreign language translation” OR “local context” OR “cross cultural test adaptation” OR “cultur* appropriate*”). No publication date limit was set, but given that digital health interventions have gained traction relatively recently, most articles were published from 2018 onward. Multimedia Appendix 1 provides the specific search strategy for each database.

### Screening

Search queries varied slightly based on the constraints of the selected databases, yielding a total of 628 papers. Abstracts of these papers were discussed in team meetings (VL, ME, AN, and IUMZ), where we applied a hermeneutic approach to honing the literature search, classifying and critically assessing the papers for sorting and screening [15]. Importantly, a hermeneutic approach is an iterative process guided by judgment, reading, interpretation, and tacking back and forth between the literature and the search approach [15]. Of the interventions reviewed in the initial search cycle, we sorted those that focused on the self-management of health, screening out those that focused on health care service delivery. As we were interested in deepening our appreciation for how interventions approached cultural adaptation, we also favored articles that presented thorough documentation of adaptation processes and considerations. A total of 113 studies were gathered in the initial screening and 2 authors (AN and IUMZ) engaged in a second cycle of iterative analysis, reading the texts in full and enriching their critical assessment of interventions using cultural adaptation. The 2 authors assessed the eligibility of the 113 studies independently by dividing the work evenly. This was carried out within the same workspace where they could readily discuss questions regarding eligibility as they arose. As the overarching criteria for paper selection were established prior, the minor questions that arose were resolved through discussion between the 2 authors with reference to the previously stated eligibility criteria. A final set of 16 studies were selected for full analysis, based on their meaningful engagement with contextual, methodological, and content-based factors in adaptation processes, with eligibility confirmed jointly by senior researcher authors (VL and HML).

### Data Extraction

The studies were analyzed for information about intervention purpose, target population, context, adaptation processes, and modifications of intervention components. All data were extracted by the lead author (AN) with oversight from senior researcher authors (VL and HML). Our approach was grounded in a qualitative mode of analysis, which combines a case-specific, detailed view with a focus on interconnectivity across cases for a better picture of the ways culture and context are conceptualized. These considerations are presented through a narrative synthesis approach [14], which enables an integrative and in-depth understanding of cultural adaptation in digital health.

## Results

### Overview

The literature presented covers both digital health interventions that have been developed without a preexisting template and those adapted from an original template. There are diverse cultural contexts represented across the studies, with some targeting racial and ethnic minority groups within high-income countries such as the United States, and others adapted for low- and middle-income countries (Table 1). The interventions range from pregnancy and parenting programs to those aimed at the prevention and treatment of mental health disorders. Moreover, there is a high representation of health literacy, health information, and health promotion interventions. Most of these are mobile health (mHealth) apps used on smartphones or tablets (12/16, 75%), while a small number are web-based interventions (4/16, 25%). By presenting diverse examples of the in-depth processes underpinning digital health cultural adaptation, we have intended to show that this is an undertaking relevant for a wide range of such interventions.

**Table 1:** Interventions featured in the review.

Study, year	Intervention	Adapted content	Models and methods
Abuwalla et al [16], 2017	Project Competent Adult Transition with Cognitive-behavioural, Humanistic, and Interpersonal Training: a web-based depression prevention intervention adapted for Arab adolescents and young adults	3 cultural domains of identity, empowerment, and expectations applied to website content	The PEN-3 theoretical framework, using 3 domains of adaptation
Alsswey et al [17], 2020	(Unnamed) app for managing medication information and delivering health information across Arab countries	Images, colors, layout, and language	Analysis of app user interface design
Bailin and Bearman [18], 2022	Parenting A to Z: a digital single-session positive parenting intervention for low-income, Latinx families in the United States	Language, persons, context, content, and metaphors	A 2-phased, experimental mixed methods design with a cultural sensitivity framework
Bender et al [19], 2016	(Unnamed) physical activity mHealth^a^ app for the Latino community in the United States	Cultural values in images and text and other culturally appropriate content; communicating information based on general literacy levels in the population	A 3-phased study exploring perceptions of images of the app; using findings to design and develop the app; testing the app for feasibility and efficacy
Black et al [20], 2018	Circle of Life: a web-based multimedia sexual health curriculum for American Indian and Alaska Native communities	Mode of intervention delivery and stories	Community-based participatory research
Doty et al [21], 2020	Padres Informados/Jovenes Preparados: mHealth app for Latinx parents of adolescents	Human-centered design principles applied to content design	Community-based participatory research approach, including surveys, interviews, and workshops across different stages
Gance-Cleveland et al [22], 2021	StartSmart: a perinatal outcomes mHealth app adapted for pregnant Hispanic women	Culturally appropriate translation of app from English to Spanish; consideration of conceptual validity of content	The Beaton process, involving 6 stages of translation
Garner et al [23], 2021	(Unnamed) A hypertension health literacy app for use in India	App content not discussed	Theories of cultural humility and social innovation collaboration
Hong et al [24], 2022	Wellness Enhancement for Caregivers: an mHealth program for Chinese American family caregivers of persons with dementia	Cultural values in translation and content; adapting for limited English proficiency and limited knowledge of health systems	Community-based, user-centered design principles
Kim et al [25], 2020	Health Club: a health promotion app for middle-aged, Korean Chinese migrant women in Korea	Content is not described	Living laboratories and intervention mapping methods
Mairs et al [26], 2020	Play Kindly: a cross-platform parenting app for Pacific and Māori families	App interface, scenario scripts, narration, and artwork; involvement of Pacific professionals in app development	Pacific and Māori research methodologies and qualitative methods to evaluate the app
Mueller et al [27], 2020	Maternal and Neonatal Technologies in Rural Areas: a neonatal game app for mothers in rural Nepal	Culturally appropriate images and audio	Co-design and cocreation
Abi Ramia et al [28], 2018	Step-by-Step: a digital mental health intervention adapted for Palestinian, Syrian, and Lebanese communities in Lebanon	Language of content; characters and illustrations; psychological concepts	Bottom-up process of adaptation using cognitive interviewing techniques
Ruvalcaba et al [29], 2019	Visit Planner: an English iPad tool for patient use before primary care visit, adapted for the Spanish-speaking Latino population in the United States	Culturally relevant concepts, images, and characters; adapting for literacy level; tackling stigma; use of peer-to-peer advising	3-step “transcreation” model (“translation” and “creation”)
Sit et al [30], 2020	Step-by-Step adapted for Chinese young adults	8 culturally sensitive elements were chosen as targets of adaptation: language, people, metaphors, content, concepts, goals, methods, and context	Ecological validity model
Smith et al [31], 2014	GetHealthyHarlem: a health literacy website for racial and ethnic minority groups in Harlem, New York	Adapting content for literacy level; short articles, interviews, and anecdotes from community members and local health professionals; features fostering local interactions	Community-based participatory research

^a^mHealth: mobile health.

**Figure 1:**
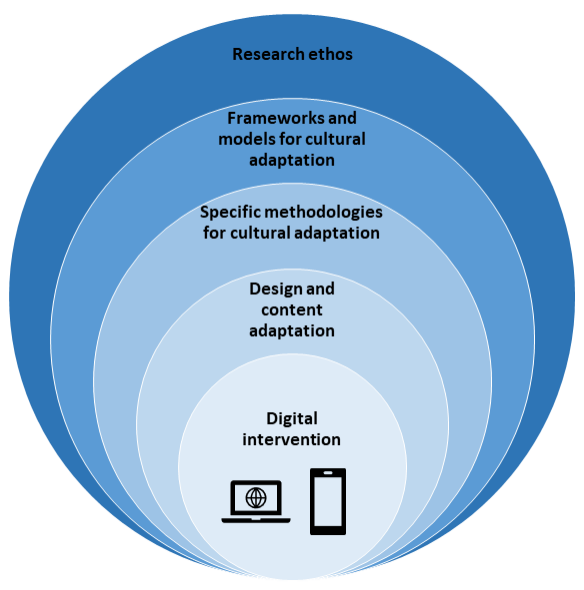
Considerations and approaches to cultural adaptation in digital health.

Figure 1 depicts the layers of considerations and approaches to cultural adaptation in digital health. The first factors to consider relate to the research ethos, including how to navigate cultural differences within the research team as well as whether the use of culturally specific research methods is recommended. Next, we explore frameworks and models applied in adaptation, with particular attention to the cultural sensitivity framework, the ecological validity model, and the PEN-3 model. The third consideration is specific methodological strategies such as participatory research approaches used for accessing cultural insights and localizing global interventions. As the final aspect, we consider specific design-related aspects of adaptation, with a specific focus on language translation and user interface (UI) design.

### Research Ethos

#### Navigating Cultural Differences Within Research Teams

Social engagement and collaboration across cultural differences within research teams and partnerships are foundational aspects of cultural adaptation. Garner et al [23] drew on the theory of cultural humility in their evaluation of the development of a hypertension health literacy app for use in India. Cultural humility can be distinguished from cultural competence in terms of being an active commitment to self-awareness and ongoing learning on the part of practitioners, rather than an end point of mastering cultural differences as implied by the term competence [32]. Garner et al [23] showed that cultural humility can be sustained within research teams through continuous reflection of individual and group interactions, the acknowledgment and mitigation of challenges, willingness to compromise and adapt perceptions and processes, as well as strengthening partnerships. Furthermore, cultural humility makes room for the identification of local cultural values that may differ from the developer’s perspective. Recognition of the importance of communal decision-making in India resulted in app design changes including a 3D animation that depicts an individual talking about hypertension with family members and friends in a rural community setting [23]. The integration of such values as communal learning into intervention development will be further discussed in subsequent sections, as it is a distinct challenge for cultural adaptation given the individualized format of most digital interventions.

#### Culturally Specific Research Methods

The literature shows that some research contexts call for the use of culturally specific methods and protocols. This is evident in the evaluation of the first parenting app designed for a contemporary Pacific audience in New Zealand [26]. Play Kindly uses gamification to teach parenting strategies for everyday behavior problems such as fights over belongings, refusing to eat, and temper tantrums. Here, culturally appropriate research involved the inclusion of Pacific professionals in the creation of the app and also drew on Pacific and Māori research methods and guidelines (ie, Kaupapa Māori research methods ensures research is conducted “by Māori, for Māori, with Māori”) [33] in the qualitative evaluation study of the app, including a welcome ceremony, food sharing, and setting the scene for the research [26]. These are framed around Pacific cultural values of communal relationships involving reciprocity, holism, and respect, while also integrating established concepts such as Teu le Vā—which involves identifying and understanding the “vā,” or spaces, between different stakeholders in Pacific research—and sharing knowledge across these spaces [26]. Notably, the conduct of evaluation research within such a cultural framework remains unique in the cultural adaptation literature.

### Models and Frameworks for Guiding Cultural Adaptation

#### Overview

Some studies in the literature use frameworks as a guide for the adaptation process, while others use adaptation models for a more systematic or staged approach for tailoring an intervention to cultural context. The extent of documented details, processes, methods, and content modifications can differ widely based on which frameworks or models are used. In subsequent sections we describe 3 of these frameworks as applied in the literature.

#### Cultural Sensitivity Framework

The cultural sensitivity framework was conceptualized by Resnicow et al [2] in the context of substance use prevention interventions. This framework is characterized by surface and deep structure adaptations and focuses on the involvement of stakeholders and end user populations to ensure cultural fit. Bailin and Bearman [18] applied this framework to enhance the customization, acceptability, and validity of evidence-based positive parenting practices presented in video format for low-income Latinx families in the United States. After conducting an initial needs assessment, researchers embedded themselves in the clinic to gather stakeholders’ input and gain a basic understanding of the procedures involved on site. Interviews with caregivers were then conducted once a prototype was made to make surface structure adaptations, including changes to the language, persons, and metaphors used in the intervention [18].

#### Ecological Validity Model

Phased approaches to cultural adaptation are common with the use of adaptation models, with one widely recognized example being the ecological validity model. This addresses 8 culturally sensitive domains in an intervention: language, people, metaphor, content (eg, cultural knowledge about values and customs), concepts (eg, treatment constructs that resonate locally), treatment goals, methods and procedures used to accomplish the treatment goals, and context [30]. Following this model, the adaptation of Step-by-Step for the Chinese young adult population in mainland China and Macao was based on a 4-phased approach [30]. The adaptation process was shaped by the lived realities of Chinese young adults concerning accepted ways of social interaction, mental health challenges experienced at this life stage, societal norms around mental health such as stigma, and content preferences based on gender. As an example, in focus group discussions, experts and laypersons suggested that the intervention would be more effective framed as a stress management tool or an academic performance tool, rather than a mental health intervention, to reduce the stigma associated with using the latter [30]. Despite drawing on the systematic approach of the ecological validity model, cultural adaptation often requires multiple iterations to ensure interventions are applicable for and relevant to potential end users. In the example of the Chinese adaptation of Step-by-Step, it was noted that the voices of more diverse young adults, including those who live with higher levels of depression would likely improve intervention acceptability [30].

#### PEN-3 Model

The PEN-3 model was developed to address the omission of culture in explaining health outcomes in existing health behavior theories and models [16]. The model has three interrelated dimensions: (1) cultural identity, (2) relationships and expectations, and (3) cultural empowerment. Each includes 3 facets that form the acronym PEN: person, extended family, and neighborhood (cultural identity domain); perceptions, enablers, and nurturers (relationship and expectations domain); and positive, existential, and negative (cultural empowerment domain). In this way, the model centralizes culture in the study of health beliefs, behaviors, and outcomes, as well as in the development, implementation, and evaluation of public health interventions [34].

By focusing on the contextual factors shaping daily life and relationships in the Arab world, the PEN-3 model enabled deep adaptation of Competent Adulthood Transition with Cognitive-behavioral, Humanistic, and Interpersonal Training, a web-based depression prevention intervention for at-risk adolescents and young adults [16]. This included addressing how the therapeutic approach can be made to fit a family-centered worldview, rather than the Western therapeutic emphasis on the individual. It also allowed norms around social relationships to be incorporated into the intervention, for example, by reducing stories that feature interactions between men and women. Moreover, the stigma around mental illness and its links to concepts of shame and religious beliefs were addressed through this model by including religious verses that promote seeking treatment for mental health disorders and discouraging labeling of patients with psychiatric conditions. The internet format is an advantage here as it offers a treatment option where no private information needs to be disclosed, thus serving the cultural value of protecting family honor and dignity [16]. This resonates with survey findings by Kamel et al [35] where psychiatrists indicated privacy, confidentiality, and security to be the most important barriers to the implementation of digital mental health interventions in Middle Eastern countries such as Egypt.

As the local adaptations of Step-by-Step and Competent Adulthood Transition with Cognitive-behavioral, Humanistic, and Interpersonal Training demonstrate, an original template can undergo substantial changes once the norms and preferences of local community members and experts are integrated through a cultural adaptation model. While not all interventions deploy a model, these may lead to more thorough adaptations of content, including language and UI design, which we focus on next [36].

### Methodologies of Adaptation

#### Methods for Accessing Cultural Insights

Cultural adaptation is a response to community-based differences in the ways health issues manifest or get addressed, making engagement with target communities a common aspect of intervention development. This is approached through diverse methods in the literature, most of which are situated in the community-based participatory research (CBPR) approach. CBPR can be understood as a research paradigm where local communities are recognized as entities with knowledge, expertise, and resources and as partners in the research process [21].

The CBPR methodology can be used in digital health programs to address issues of access and trust in health for racial and ethnic minority communities [21]. The concept of trust is significant as it relates to both socioeconomic circumstances and manifests in culturally specific ways. This is evident in the study of an mHealth app based on an in-person parenting program aimed at reducing substance use among Latinx youth. The app was developed using iterative human-centered design processes integrating feedback from stakeholders and end users and was informed by The Centre for eHealth Research and Disease Management road map for designing digital health technology [21]. This approach aided in building trust through endorsing and centering the knowledge of community stakeholders, prevention program facilitators, and parents in app development and introducing the critical analysis of cultural needs [21]. Through the CBPR process, community facilitators of the program became cocreators of content for the mobile app version, which was then validated through interviews with parents. The conduct of participatory research in the language of participants here and in other examples ensures essential information is not lost in translation, while also saving time and resources [37]. The resulting design adaptations included a minimalist esthetic, clear navigation, structural consistency, a reduction in the length of videos, and inclusion of transcripts, as well as addressing internet connectivity issues, all of which are aimed at ensuring accessibility for the audience.

The multimethod approach of “living labs” is another way to understand and address the health needs of vulnerable communities [25]. In the development of a health promotion app for middle-aged, Korean Chinese migrant women, Kim et al [25] applied core principles derived from the living laboratories approach, namely user and stakeholder participation; basis in real-life settings; and cocreation to identify stakeholder needs, set objectives, identify relevant intervention strategies, and consider design options. The intervention was designed to be culturally appropriate by virtue of attending to the challenges faced by this demographic in Korea, including experiences of social isolation alongside language barriers and changes in socioeconomic status due to migration. Moreover, the phrase “cultural adaptation” was also thought to apply to users who were provided with information about life in Korea to encourage a greater sense of belonging [25]. In this sense, through the means of tailored health promotion, the intervention sought to address ways users can also culturally adapt.

#### Participatory Methods in Localizing Global Interventions

While the interventions discussed thus far were originally developed for a local context, others have been developed as scalable templates for a global audience and later adapted for local contexts using participatory methods. A major example of this is Step-by-Step, a transdiagnostic and guided digital mental health intervention that is supported by an app-based delivery modality, targeting common mental health disorders such as depression. The intervention was developed through a top-down process involving the World Health Organization; government bodies and other large organizations as a global public good with the intervention content (eg, stories, illustrations, and videos); guidance model; and mobile app being culturally adaptable [28,30,38-40]. CBPR methods such as cognitive interviewing [4] and focus group discussions have been used in different cultural adaptations of the intervention to make its content relevant for local communities. The involvement of local stakeholders in the adaptation process is crucial for identifying and reworking concepts derived from Western science used in the intervention, namely those around mental illness and mental health. Local knowledge of the influence of contextual factors on the issues explored in the intervention means the target audience is more likely to see their experiences reflected in the intervention, thus allowing for the relevant issues to be addressed more effectively.

Indeed, the Lebanese adaptation of Step-by-Step highlights how mood disorders are affected by social, economic, and health challenges in this content [28]. The explanation of depression symptoms was changed to reflect local understandings of how depression is experienced: maintaining daily activities and work but displaying higher levels of anger, irritation, and anxiety. Furthermore, clinical terms such as “mental illness” were not used in the intervention stories, rather terms such as “distress” or local idioms such as “tired psyche” were used instead [28]. The role of faith and religion in helping people deal with mental difficulties was integrated into the activities after adaptation. Finally, gendered stereotypes were also tempered to depict cultural norms as recommended by local participants. Specifically, some changes were made to challenge the stereotypical depiction of women as submissive and expressing distress through crying or the depiction of men as expressing distress through anger; however, other gendered elements were maintained to ensure the content resonated with users. These modifications align with observations by Kamel et al [35] who stated that before the use of Western interventions in the Middle East, it is crucial to consider incompatibilities around gender-related norms, public awareness, and stigma of mental disorders, as well as language and presentation of distress.

### Intervention Design and Content Adaptations

#### Culturally Adapted Translation

Language translation is a crucial component of cultural adaptation in digital interventions, as language discrepancy and cultural differences in understandings of health or illness often pose barriers to engagement. In cases where an intervention is adapted for a new cultural context, a literal translation can result in poorly constructed sentences, mistranslations, and failure to capture the metaphors and idioms within a language [36]. The literature demonstrates that culturally adapted translation can avoid these by involving processes such as forward and back translation, as well as reviews and syntheses of different versions. In Textbox 1, we provide an example of this by summarizing the Beaton process of translating and adapting StartSmart, a perinatal outcomes app helping pregnant women screen for risk and protective factors through a self-administered questionnaire [22].

**The Beaton process of culturally adapted translation.**

**Stage and process**

- 1: Forward translation of the screening instrument and patient education materials by 2 bilingual translators.
- 2: Synthesis of translations through consensus by translators and the primary investigator.
- 3: Back translation into English by 2 different translators with English as their first language who did not see the original documents.
- 4: An expert committee review conducted with particular attention to capturing the nuances of the Spanish language. Consensus reached on the pretest version.
- 5: The app was pretested through 17 interviews with patients, and a focus group discussion with providers focused on usability and acceptability.
- 6: Appraisal of StartSmart carried out to determine whether the Beaton process was followed to translate and culturally adapt the system and consider refinements to the technology.

The “transcreation” process is another example of culturally adapted translation achieved through hiring research team members such as health educators and cultural brokers with appropriate linguistic and cultural expertise, careful assessment of intervention elements for their potential cultural significance, and iterative testing and assessment of the resulting changes [29]. This approach is particularly relevant when cultural adaptation predominantly centers on content wording and visual modifications. For example, a core cultural construct that impacts health-seeking behavior in the Latino context is “familismo” or family. This was embedded in the content of an mHealth intervention by changing the app’s menu wording, such as “Stress at Home or Work” to “Mi Familia” or my family [29].

#### Adaptation of UI Design

The UI is the point of contact and communication between users and digital interventions and is greatly emphasized by designers in relation to user experience and user acceptance [17]. UI includes the display screens and the way through which a user interacts with an app or website, such as voice- or touch-enabled features, multimedia graphics, and others. In this context, images are more than mere objects but rather cultural artifacts that represent values, ethos, or ideologies [17]. Indeed, the visual depiction of cultural values can be an effective way to increase engagement. To that end, familial cohesion and social support were centered visually in a low-text physical activity app designed for the Latino community, through the depiction of family members, friends, and peers exercising together during culturally relevant physical activities such as dancing and football [19]. Importantly, Alsswey et al [17] found a strong correlation between culturally adapted UI design and app use, in that users felt more confident when navigating technology that reflects familiar elements reflected in images, colors, language, and layout.

The principles of cocreation and co-design are vital for achieving these ends. Mueller et al [27] define cocreation as collective creativity and co-design as the application of this principle across the design process, which extends decision-making and stakes in the end product to a broader group. In the development of their gamified mHealth intervention for a low-literacy adult audience in rural Nepal, UI features such as pictograms were co-designed with in-country partners and artists and further refined through field evaluations with end users [27]. The attention to detail in the co-designed pictograms (eg, symbolic clothing colors representing marital status) was described as a crucial aspect of intervention localization [27].

#### Adapting Intervention Format

Establishing cultural relevance around values is not always clear cut, particularly when it comes to factors such as intervention format and the ways new knowledge is presented to users. In an evaluation study of the gamified intervention Play Kindly discussed previously, Mairs et al [26] pointed out disagreements among participants about how culture specific the app should be based on how strongly the person identified with their culture. This can be characterized by differences between a participant who believes parenting is not shaped by culture and one who may identify cultural influences in parenting practices. While the Pacific theme of the UI and the real-life scenarios were widely accepted by users, the approach of providing parenting information via a virtual avatar coach (referred to as “the educator”) stood out as a point of contention by leading some participants to feel as though they were making parenting mistakes. The gamified format of the app also faced mixed reactions given the rule of having to answer correctly to progress through the game, which similarly impacted users’ view of themselves as parents [26]. These factors demonstrate that the mode of information delivery, and the overall format of an intervention can be major drawcards for user engagement if aligned with cultural beliefs.

Despite the advantages of digital health interventions regarding accessibility and cost-effectiveness, the impersonal technologies can be culturally inappropriate in cultures where knowledge has traditionally been transmitted in communal settings. This is particularly significant in the context of coming of age and sexual health, which are seen as a community responsibility among American Indian and Alaska Native people [20]. Community partners involved in the development of a web-based, multimedia sexual health curriculum highlighted the need for the interpersonal approach of traditional knowledge sharing to be reflected in the digital mode of intervention delivery [20]. As a response, a hybrid version of Circle of Life was created, combining web-based lessons with class-based activities and discussions. However, contextual factors related to poverty had direct consequences for engagement, such as logistical challenges, poor internet, and low class attendance. Therefore, the intervention had to be made flexible enough so that it was responsive to local circumstances according to the discretion of facilitators, demonstrating that cultural adaptation must also integrate socioeconomic context to ensure accessibility.

## Discussion

### Overview

The methodologies, models, and content adaptations we have examined demonstrate the wide range of approaches to cultural adaptation. It is evident that while an adaptation framework or model is not a necessary prerequisite for adaptation, an iterative process with multiple stages of intervention development, testing, and refinement is essential particularly when participatory research methods are used. The extent of community participation and the depth of cultural adaptation tends to be based on a range of factors such as intervention goals, research questions, processes, and the context of intended users. Some projects also include key stakeholders representing governmental and nongovernmental organizations, as well as other local experts as part of participatory research.

The involvement of a broad range of participants can lead to more successful adaptations by adding important insights for content development and implementation [4]. This is pertinent given that consensus does not always exist around cultural relevance within a community, as people may have different opinions on values and norms [26]. Appreciation of cultural heterogeneity within cultural groups is vital to avoid insensitive material in interventions. This can be ensured by incorporating multiple perspectives that appeal to a broad spectrum of the target population [2]. When a wide scope of coverage is intended, common factors must be deduced based on key themes and patterns identified in research, hence why the inclusion of cultural expertise can be vital. For instance, in cases where one language is shared across different settings (eg, Spanish), it may make sense to use common activities and examples and provide neutral recommendations to enable an intervention’s applicability across different countries [41].

### Surface and Deep Structure Adaptation

Our review highlights that adaptation occurs on a spectrum, ranging from surface to deep level adaptations. Specifically, surface level adaptations are often integrated in the UI, involving components such as labels and illustrations. As demonstrated in many interventions in this review, such components of the UI can affect the level of usability and engagement based on whether its elements resonate with the preferences of an audience as influenced by cultural context [17]. It is essential to integrate user feedback to ensure different components in the UI are culturally appropriate, and the layout is easy to navigate for different digital literacy levels. Deep adaptation goes beyond the visual aspects of an intervention or the literal translation of its content to include culturally specific perspectives on major concepts in an intervention. This is especially pertinent in relation to interventions based on culturally sensitive topics such as mental illness, as evidenced by the integration of local understandings of mental health disorders by Ramia et al [28].

Deep adaptation is necessary if case studies and stories are to reflect the cultural characteristics of relationships, such as gender norms or the importance of family and the local community for personal health [16,29]. Furthermore, collective knowledge sharing and learning is a recurring cultural value that can impact user engagement, as it challenges the conventional focus on the individual in digital interventions. Therefore, deep cultural adaptation also involves the integration of such values in intervention design. This approach to adaptation requires a deeper level of cultural sensitivity as it reframes intervention components that may have been previously assumed to be culturally neutral and not in need of adaptation.

When decisions must be made by researchers and developers between surface and deep structure adaptation, we recommend considering the distinction between literal translation and culturally appropriate translation as a guide. Spanhel et al [7] grouped all language translations as a surface structure adaptation, but we suggest that culturally adapted translation, which includes locally specific idiomatic expressions and metaphors, can be classified as deep structure adaptation. Culturally appropriate translation not only reduces the likelihood of mistranslations but also enables different perspectives and values around concepts to be presented in the content [36]. Deep translation draws on cultural expertise and can involve multiple stages including back translation, synthesis of different translations, and consensus on the choice of translation [22]. This is a vital aspect of cultural adaptation given that digital interventions often rely on text to convey information. If text is convoluted or does not reflect local language norms, it can swiftly discourage user engagement.

Importantly, the notion of culture in adaptations of digital interventions is primarily about the familiarity, relatability, and accessibility of an intervention for users in both cases of surface and deep adaptation. This distinction around depth of cultural sensitivity and adaptation reveals that cultural adaptation cannot be prescribed beyond general guidelines, but rather must be based on contextual circumstances around an intervention.

### The Intersection of Socioeconomic and Cultural Factors

Cultural adaptation means reconceiving how we think about interventions and their interactions with context. May et al [10] have conceptualized context as a *process* rather than a *place*, suggesting that the context in which intervention implementation occurs requires ongoing work to hold together and advance. Cultural adaptation enables analysis of the ways digital interventions interact with particular local contexts [40]. This involves not only actively integrating key elements of context into interventions but also exploring the ways these are implemented as users and stakeholders may themselves engage in processes of cultural adaptation once interventions are rolled out [42].

The studies examined in this review show that culture interacts dynamically with other contextual factors such as economic conditions and social systems that impact the accessibility and sustained use of interventions. Low literacy resulting from poverty or migration is a case in point. Many of the interventions featured have placed great emphasis on ensuring content is accessible by simplifying the UI design, reducing text length, and avoiding jargon words [19,21,24]. Other socioeconomic challenges such as isolation and stigma are also addressed as part of cultural adaptation, for example, by including features that encourage social interaction in the community [31], or offering alternative ways to discuss stigmatized topics [16]. Barriers to digital sources due to limited technological knowledge, mobile data costs, and the lack of devices are further contextual issues related to accessibility that are addressed alongside culture in these adaptations [41]. Given that digital interventions are often developed as a response to health challenges linked to socioeconomic factors, cultural adaptation is more likely to be effective when addressing culture outside of a vacuum. Detailed documentations of cultural adaptation create further opportunities to tease out the dynamic connections that constitute context.

### Limitations

Given that this search had no specified date range, the literature surfaced during this review was very comprehensive in nature, enabling an in-depth exploration of the considerations and methods for cultural adaptation in digital health. It is important to note that the search was performed in November 2022 meaning that studies of cultural adaptation conducted more recently were not included in this narrative synthesis. However, in keeping with a hermeneutic approach described by Boell and Cecez-Kecmanovic [15], we reached saturation in terms of understanding how concepts of culture and context featured in design and develop processes and interventions; therefore, extending the search to the present would not add to the already rich themes.

### Conclusions

Digital health interventions offer potential for direct, cost-efficient, and convenient ways to address local and global health challenges. Cultural adaptation is a key means of increasing the engagement of target communities so that this potential may be realized. While cultural adaptation has been critiqued as limiting who can engage with an intervention in terms of ethnicity, age, or income [18], this narrative review shows that adapted interventions are usually aimed at addressing issues in public health systems where preexisting interventions are failing to reach communities. This is true both globally in terms of the digital health gap in low- and middle-income countries and within countries with regard to marginalized communities and segments of the population such as youth and caregivers [43].

When embarking on cultural adaptation, decisions around the level of adaptation must consider the purpose of the intervention, complexity of its content, and the context of implementation. In this regard, we argue that wider project considerations such as budget and timelines should be tailored for adaptation, rather than determining adaptation decisions. Moreover, establishing what is culturally relevant is not straightforward. We suggest culturally humble approaches that use the involvement of a broad range of participants, experts, and other stakeholders as these can lead to more successful adaptations by sparking important insights for content development and implementation [4]. Where feasible, long-term research (eg, ethnographic fieldwork) is recommended as a way of gaining deeper understanding of local contextual meanings and conditions. The participatory engagement of stakeholders can be best harnessed when insights are iteratively fed back across all stages, from development to validation and evaluation phases.

Rigorous studies of cultural adaptation in the digital context remain scarce. The field of digital health would benefit from more studies documenting processes of cultural adaptation and implementation, including feasibility and effectiveness, as well as evaluation to establish impact. Similarly, more studies are welcomed that interrogate the relationships between culture and the broader context in which people interact with digital resources. This research is critical as the impacts of interventions are influenced by the contexts around implementation and use.

## Appendix

Multimedia Appendix 1 Database search strategy.

## Abbreviations

CBPR: community-based participatory research
mHealth: mobile health
UI: user interface

## Data Availability

Data sharing is not applicable to this paper as no data sets were generated or analyzed during this study.
