# Supplementary material for: Cultural and Contextual Adaptation of Digital Health Interventions: Narrative Review
Source: J Med Internet Res. 2024 Jul 9;26:e55130. doi: 10.2196/55130 (PMC11267096; doi:10.2196/55130)
Supplement: Multimedia Appendix 1 [file jmir_v26i1e55130_app1.docx]

**Multimedia Appendix 1. Database search strategy**

APA PsycInfo

APA PsycInfo <1806 to April Week 5 2024>
1 (("digital intervention" OR "digital health" OR "mobile health" OR "mhealth" OR "Electronic Health Services" OR "ehealth" OR "telemedine" OR "telehealth" OR "parenting app" OR "parent* app") AND ("sociocultural factors" OR “cultural sensitivity" OR "cultural adaptation" OR "foreign language translation" OR "local context" OR "cross cultural test adaptation" OR "cultur* appropriat*")).af.

2 limit 1 to (peer reviewed journal and english language)

SCOPUS

((TITLE-ABS-KEY("digital intervention" OR "digital health" OR "mobile health" OR "mhealth" OR "ehealth" OR "telemedine" OR "telehealth" OR "parenting app" OR "parent* app") AND TITLE-ABS-KEY("sociocultural factors" OR "cultural sensitivity" OR "cultural adaptation" OR "foreign language translation" OR "local context" OR "cross cultural test adaptation" OR "cultur* appropriat*")))

CINAHL

"( "digital intervention" OR "digital health" OR "mobile health" OR "mhealth" OR "Electronic Health Services" OR "ehealth" OR "telemedine" OR "telehealth" OR "parenting app" OR "parent* app") AND ("sociocultural factors" OR cultural sensitivity" OR "cultural adaptation" OR "foreign language translation" OR "local context" OR "cross cultural test adaptation" OR "cultur* appropriat*" )"

Limiters - English Language; Peer Reviewed; Research Article; Exclude Pre-CINAHL; Exclude MEDLINE records; Human;

Ovid MEDLINE(R)

Ovid MEDLINE(R) ALL <1946 to May 03, 2024>

1 (("digital intervention" or "digital health" or "mobile health" or "mhealth" or "Electronic Health Services" or "ehealth" or "telemedine" or "telehealth" or "parenting app" or "parent* app") and ("sociocultural factors" or "cultural sensitivity" or "cultural adaptation" or "foreign language translation" or "local context" or "cross cultural test adaptation" or "cultur* appropriat*")).af.

2 limit 1 to english language
